# Synergistic Neuroprotective Effects of a Plant‐Derived Alkaloid and Insulin‐like Growth Factor‐1 on SH‐SY5Y Cells: Targeting the Wnt/β‐Catenin Pathway in Alzheimer's Disease

**DOI:** 10.1002/alz70859_096869

**Published:** 2025-12-25

**Authors:** Prajjwal Sharma

**Affiliations:** ^1^ Central University of Punjab, Bathinda, Punjab India

## Abstract

**Background:**

Alzheimer’s disease (AD) is the leading cause of dementia, affecting millions worldwide and necessitating innovative therapeutic strategies to halt its progression. Traditional herbal compounds are increasingly studied for their therapeutic potential and lower side effects. This study investigates the combined effects of a natural alkaloid and Insulin‐like Growth Factor 1 (IGF‐1) on amyloid‐beta (Aβ)‐induced neurotoxicity in SH‐SY5Y cells, exploring their potential as a novel treatment for AD.

**Method:**

SH‐SY5Y cells were cultured in 1:1 DMEM and F12 medium with 10% fetal bovine serum, antibiotics, and glutamine at 37°C with 5% CO2. Aβ‐induced neurotoxicity was established, while a control group was maintained without Aβ exposure. The half‐maximal inhibitory concentration (IC50) of the natural alkaloid was determined, identifying specific concentrations for subsequent testing. IGF‐1 was similarly tested to determine an optimal dose. Cells were pre‐treated with these natural alkaloid and IGF‐1 doses before Aβ exposure to evaluate their neuroprotective effects. Assays performed included MTT for cell viability, DCFDA for ROS, TMRM for mitochondrial ROS, and measurements of MDA, GSH, SOD, and catalase. Apoptosis was assessed using Annexin V and acridine orange staining. Neuronal growth and health were evaluated with NeuN and cresyl violet staining, while molecular markers such as GSK‐3β and β‐catenin activities were analyzed.

**Result:**

Natural alkaloid and IGF‐1 treatment significantly improved cell viability in Aβ‐exposed SH‐SY5Y cells, underscoring their neuroprotective effects. Both treatments effectively reduced ROS levels and apoptosis, indicating a reduction in Aβ‐induced oxidative stress and cell death. Additionally, there were marked improvements in neuronal markers and favorable modulations in GSK‐3β and β‐catenin activities, highlighting the potential of this combined therapeutic approach to address key AD pathophysiological mechanisms.

**Conclusion:**

This study highlights the significant neuroprotective potential of combining a natural alkaloid and IGF‐1 against Aβ‐induced toxicity, presenting a novel and promising therapeutic strategy for AD. Future research should aim to elucidate the detailed mechanisms underlying these protective effects and confirm their efficacy in in vivo models.

